# Policies and practices to attract, retain, support, and reskill health and care workers during the COVID-19 pandemic and future workforce development in Thailand

**DOI:** 10.7189/jogh.13.03046

**Published:** 2023-11-17

**Authors:** Vichai Tienthavorn, Wanicha Chuenkongkaew, Vasuton Tanvatanakul, Sukjai Charoensuk, Pisit Poltana, Phayong Thepaksorn

**Affiliations:** 1 President Office, Praboromarajchanok Institute, Royal Thai Ministry of Public Health; 2 Boromarajonani College of Nursing, Chonburi, Faculty of Nursing, Praboromarajchanok Institute, Royal Thai Ministry of Public Health; 3 Boromarajonani College of Nursing, Suphanburi, Faculty of Nursing, Praboromarajchanok Institute, Royal Thai Ministry of Public Health; 4 Sirindhorn College of Public Health, Trang, Faculty of Public Health and Allied Health Sciences, Praboromarajchanok Institute, Royal Thai Ministry of Public Health

**Figure:**
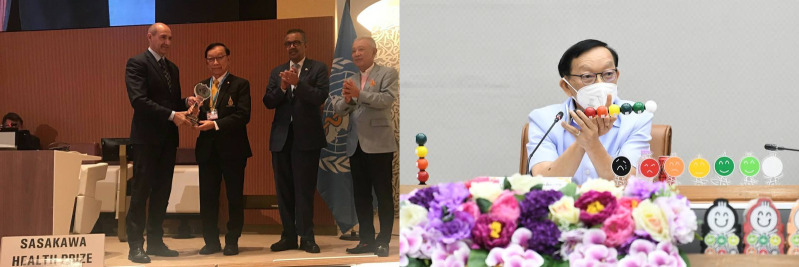
Photo: Professor Vichai Tienthavorn, President, Praboromarajchanok Institute, Ministry of Public Health, Thailand established the perspective and direction of the research and presented conceptualised the viewpoint of this project. He has been named the recipient of the World Health Organization’s prestigious Sasakawa Health Prize 2023 for his work on thalassemia and other non-communicable diseases such as thalassemia, diabetes and hypertension. Source: From authors’ own collection, used with permission.

The policy of the Ministry of Public Health (MoPH) of Thailand successfully contained the coronavirus 2019 (COVID-19) pandemic. Its comprehensive duty is to lead the emergency response to prevent, contain, and control health challenges ranging from the national agenda to primary care in the communities. Key elements for tackling burdens include building the health workforce capacity and crafting public health policy that facilitates the capacity to respond to this pandemic [1,2].

To enhance the capacity of health care workers to respond to the pandemic, Praboromarajchanok Institute (PBRI) is a higher educational institution whose main mission is to produce and develop human resources according to the needs of the MoPH [3]. PBRI has both implemented new policies and strengthened current policies to attract, retain, support and re-and up-skill health care workers during COVID-19 in Thailand. PBRI promotes community health through its affiliated colleges, which are located in every region of the country. PBRI also has close relationships with the communities it serves, which are akin to family. PBRI has been fully integrated at the primary health care level, through its community and health promoting hospitals that provide first-contact services available to everyone [4,5].

## POLICY INTERVENTIONS

To make health systems more resilient to disaster preparedness and crises, PBRI plays a major role in producing health science graduates at both under- and post graduate levels. These graduates serve as health personnel in rural areas, and make up more than half of the health workforce in the country [6,7]. The MoPH and PBRI have greatly contributed to solving the national crisis on COVID-19 [1,6,8,9]. It promoted proactive action to prevent and monitor the spread of COVID-19 through village health volunteers (VHVs). VHVs use a smartphone application tool for surveillance of the COVID-19 epidemic. PBRI also now offers additional instruction for VHVs in nursing assistant and public health assistant program (1 year course).

To support and built health workforce capacities, the Department of Disease Control mobilized experienced medical personnel and epidemiologists from provinces with any surplus capacity [10]. The MoPH deployed doctors, nurses, and health personnel to support quarantine sites. Local administrations mobilized one million VHVs to boost the capacity of the national surveillance system. In addition, the newly recruited VHVs received 43 hours of public health ministry-funded training. Because they typically shared the same dialect, religion and sociocultural practices of local communities, VHVs were invaluable in reaching local populations amidst challenging circumstances [8].

The Department of Health Service Support, MoPH is responsible for controlling and regulating systemic health services and in order to proceed in accordance with the situation. To cope with COVID-19, a quarantine system included 4 different styles, including 1) Alternative State Quarantine (ASQ), 2) Alternative Hospital Quarantine (AHQ), 3) Golf Quarantine, and 4) Wellness Quarantine. Village health volunteers (VHVs) were front-line personnel working on control, prevention, and surveillance of disease. They also came into contact with patients infected with COVID-19. VHVs played major roles in monitoring the spread of the disease in the community, following up on target populations who have travelled from Bangkok and its vicinity, as well as groups that travelled from abroad. They (n = 591 058 VHVs) have conducted in-person home visits to advise and educate about 7 424 625 people about the prevention of COVID-19 so far. These extensive efforts contributed to Thailand being internationally ranked 4th in the world in controlling COVID-19 [8,9].

PBRI plays a role in solving the national crisis on COVID-19. It involves with stakeholders and parties in community with proactive action to prevent and monitor the spread of COVID-19. Efforts also included using the application tool for surveillance of the COVID-19 epidemic. They also recruited 6000 VHVs for training in the nursing assistant and public health assistant program.

## OUTCOMES

To address uneven geographical distribution of health care workforce, the MoPH applied comprehensive interventions to increase the number of health care workers in rural areas. Interventions included increasing training capacities and recruiting students from underrepresented rural communities through assigned high schools with memorandums of understanding (MOUs) under the Ministry of Education.

VHVs and subdistrict health center personnel have a good understanding of local community dynamics. Thus, they can efficiently contribute to the prevention and control of COVID-19. The VHVs help the public health personnel in several roles, including 1) assisting with vaccination and contributing to the performance reporting system, 2) knowledge of preventive vaccination, and 3) using digital technology of Line Official Account technology “MorProm App,” which provides a follow-up surveillance platform. The total, cumulative number of proactive case findings increased to 12.6 million home visits and 834 000 COVID-19 cases by April 2020. VHVs greatly contributed to the prevention and control of COVID-19 in Thailand. Rapid and effective implementation of prevention and control of infectious disease pandemics at a national scale was effective due to the contribution of VHVs and involved parties. Additionally, the health risk communication network can effectively fill gaps in information from the VHVs.

The low number of COVID-19-positive cases in Thailand indicates the robustness of Thailand’s health care system in responding to public health needs and emergencies. VHVs have played a pivotal role in detecting and reporting COVID-19-positive cases in communities across the country. The MoPH manages 1.04 million VHVs across the country in both urban and rural areas. The timely mobilization of Thailand’s VHVs, who are educated, well-trained and experienced in infectious disease surveillance, enabled the robust response of the country’s health services during the COVID-19 pandemic. They act as a representative and proactive surveillance gatekeeper of the community in the response to the COVID-19 pandemic [11].

To address social interventions, build trust, and ensure compliance, the government set up the Centre for COVID-19 Situation Administration for communicating risk and engaging communities in its daily broadcast on all media channels. A shortage of specialists, in particular intensive care nurses and critical care experts, became critically evident at the peak of the epidemic. The MoPH closely monitored the pandemic at a provincial level and managed the reallocation of resources. It deployed special, on-the-job training. In provinces with a high case load and a critical shortage of health care workers, medical teams were mobilized from other provinces [8].

VHVs devoted themselves to their work by conducting surveillance of the health care needs of their community and developing a close relationship with their assigned populations. The time that they spent varied from a few hours per week to long shifts during busy periods, such as the COVID-19 pandemic surges. VHVs received compensation and benefits from the government toward expenses, such as reimbursement of travel costs, discounted hospital costs for their families, etc. [8]. To strengthen and scale up the health care workforce, PBRI provided a special enrollment program for the offspring of VHVs to become health care students by signing MOUs with high schools across the country to enable equal access to health professional education in the community.

To prepare VHVs to respond to COVID-19 on a large scale across the country, the MoPH delivered online courses and local health officers provided in-person training for the VHVs [8,12]. The training included basic knowledge of COVID-19, how to communicate and stay safe, how to identify and monitor members of the community at high risk, especially high potential risk groups, older people or those with chronic illness such as cardiovascular disease, diabetes, or hypertension. The close relationship between the VHVs and populations of their rural communities enabled the smooth functioning and strengthening of disease surveillance.

## CONCLUSIONS

To enhance the capacity of health care workers to respond to the pandemic, the MoPH and PBRI are working closely and play a vital role to produce and develop human resources according to health care system in responding to public health responses and emergencies. They have both implemented new policies and strengthened current policies to attract, retain, support and re-and up-skill health care workers during COVID-19 pandemic in Thailand. They have been fully integrated at the primary health care level, through its community, VHVs and health promoting hospitals that provide first-contact services available to everyone.
